# COIBar-RFLP Molecular Strategy Discriminates Species and Unveils Commercial Frauds in Fishery Products

**DOI:** 10.3390/foods11111569

**Published:** 2022-05-26

**Authors:** Anna Maria Pappalardo, Marta Giuga, Alessandra Raffa, Marco Nania, Luana Rossitto, Giada Santa Calogero, Venera Ferrito

**Affiliations:** 1 Department of Biological, Geological and Environmental Sciences, Section of Animal Biology “M. La Greca”, University of Catania, Via Androne 81, 95124 Catania, Italy; pappalam@unict.it (A.M.P.); marta.giuga@phd.unict.it (M.G.); alessandra.raffa92@gmail.com (A.R.); marco.nania54@gmail.com (M.N.); lunarossa92@gmail.com (L.R.); giadacalogero@gmail.com (G.S.C.); 2 Institute for the Study of Antropic Impact and Sustainability in the Marine Environment, IAS-CNR, 91021 Trapani, Italy

**Keywords:** COIBar-RFLP, molecular traceability, fish species authentication

## Abstract

The DNA analysis is the best approach to authenticate species in seafood products and to unveil frauds based on species substitution. In this study, a molecular strategy coupling Cytochrome Oxidase I (COI) DNA barcoding with the consolidated methodology of Restriction Fragment Length Polymorphisms (RFLPs), named COIBar-RFLP, was applied for searching pattern of restriction enzyme digestion, useful to discriminate seven different fish species (juveniles of *Engraulis encrasicolus* and *Sardina pilchardus* sold in Italy as “bianchetto” and *Aphia minuta* sold as “rossetto”; icefish *Neosalanx tangkahkeii*; European perch, *Perca fluviatilis* and the Nile Perch, *Lates niloticus;* striped catfish, *Pangasianodon hypophthalmus*). A total of 30 fresh and frozen samples were processed for DNA barcoding, analyzed against a barcode library of COI sequences retrieved from GenBank, and validated for COIBar–RFLP analysis. Cases of misdescription were detected: 3 samples labeled as “bianchetto” were substituted by *N. tangkahkeii* (2 samples) and *A. minuta* (1 sample); 3 samples labeled as “persico reale” (*P. fluviatilis*) were substituted by *L. niloticus* and *P. hypophthalmus.* All species were simultaneously discriminated through the restriction pattern obtained with *Msp*I enzyme. The results highlighted that the COIBar-RFLP could be an effective tool to authenticate fish in seafood products by responding to the emerging interest in molecular identification technologies.

## 1. Introduction

Two important topics concerning the fisheries sector, have been focused in the legislation of the European Union (EU). On one hand the EU has adopted a common fisheries policy to promote the conservation and the sustainable use of the fishery resources, on the other hand regulations have been laid down to protect consumers from illegal practice. These two seemingly unconnected problems actually influence each other. Indeed, the depletion of fish stocks in the Mediterranean Sea has led to regulate catches and landing of fishery products in the framework of an effective and responsible management of the resources by the Member States (EU Regulation No 1241/2019). For that reason, Total Allowable Catches (TACs) are set annually for most fish stocks on the basis of scientific advice on their conservation status; TACs are then shared between EU countries in the form of national quotas. For some particularly depleted species, a closure period in the entire area of catch has been also established. Furthermore, attention has been paid to avoid the excessive catches of undersized specimens through the protection of certain areas where juveniles congregate (Council Regulation No 1967/2006). Against these important and very useful initiatives aimed to improve the sustainability of fishing, the practice of commercial fraud has increased, also encouraged by the global increase of demand of fishery products. Furthermore, the risk of adulteration of fishery products has increased dramatically as a result of the COVID-19 pandemic and the BREXIT [1]. The most common commercial fraud is based on replacing fish of value with species of lower commercial value, in transformed products, for an evident economic gain [2,3,4,5]. However, in addition to the economic damage, this illegal practice entails serious threats to the health of consumers and to the conservation of endangered species [6,7,8,9,10,11]. Accordingly, the need to unveil commercial frauds to protect consumers has become urgent, worldwide highlighted and pursued through regulations laid down by control bodies at international and national level. The regulations of the European Parliament aimed to fight seafood commercial frauds was focused on labelling rules. In particular, the EU Regulation No 1379/2013 on the common organization of the markets in fishery and aquaculture products, made mandatory to indicate on the label “*the commercial designation of the species and its scientific name; the production method; the area where the product was caught or farmed, and the category of fishing gear used in capture of fisheries*…”. However, it should be noted that the aforementioned regulations have not led to the disappearance of commercial fraud [12].

Another incontrovertible fact is that the DNA analysis is the best approach to unveil the illegal practice of species substitution, a fraud difficult to identify in all cases where the morphological identification of the species is impracticable because of food processing (i.e., ready to eat products or frozen fillets). Several molecular marker and methodologies have been proposed to be applied for authentication of species [13] and the mitochondrial genes (Cytochrome Oxidase I, cytochrome b, control region, 16SrDNA) are the most used molecular markers [14]. However, while the barcoding methodology based on sequencing of the mitochondrial Cytochrome Oxidase I gene (COI) has become the marker of choice for identifying animal species and fish species in particular [15,16], the other mitochondrial genes and the control region in particular, have been most used for studies of population genetic structure [17,18,19,20,21,22,23,24,25,26,27,28]. Indeed, after almost 20 years since Hebert et al. [29] proposed the COI as a tool for a global bioidentification system for animals [15,30,31,32], COI barcode is being worldwide used to unveil commercial seafood fraud based on mislabeling [2,33,34,35,36,37,38]. In addition, the barcoding methodology which is reliable, accurate and reproducible, has been improved to make it time and cost-effective and then useful for routine screening by industry and for food forensics investigations. Implementation has been obtained by coupling the barcoding with classic and new generation methodologies, such as the Random Fragment Length Polymorphisms (RFLP), High Resolution Melting Analysis (HRMA) and species-specific Real Time Polymerase Chain Reaction (PCR) [39,40,41]. For several years, the RFLP methodology based on the digestion of restriction enzymes applied to the COI sequences, renamed COIBar-RFLP [42] has been successfully used for species authentication in processed seafood [11,43,44,45]. In the present investigation, this combined method will be used to unveil fraudulent practices of species substitution in seafood products sold as “bianchetto” (juveniles of *Sardina pilchardus* and *Engraulis encrasicolus*) and “rossetto” (*Aphia minuta*), the so-called “special forms of fishing” prohibited by European regulations with the possibility of exemptions (Council Regulation 1967/2006) and those sold as fillets of European perch (*Perca fluviatilis*) a species of high commercial interest. The aim is to demonstrate that by coupling two consolidated methodology, the PCR-RFLP worldwide used in the laboratory and the DNA barcoding based on the COI sequences (available in a very high number, in Barcode of Life Data Systems (BOLD) and GenBank large public gene sequence databases) could be a cost-effective and standardized solution for large-scale screening of seafood species authentication.

## 2. Materials and Methods

### 2.1. Sampling

The seafood products analyzed in this study include the juveniles of *E. encrasicolus* and *S. pilchardus* sold in Italy as “bianchetto” and *A. minuta* sold as “rossetto”, they are consumed in several regions of Italy and are part of the Italian culinary culture since 1800; the icefish *Neosalanx tangkahkeii*, introduced in the Italian fish market; the European perch, *P. fluviatilis*, a freshwater species living in the northern Italian rivers and *Lates niloticus*, the Nile Perch, whose import into Italy has increased in the last decade. Fresh and frozen samples of examined fish products were acquired in 2020 and 2021 from local fish markets and supermarkets of Southern Italy for a total of 30 samples. All samples, preserved at room temperature in 1.5 mL labeled tubes filled with 95% ethanol, were processed for DNA barcoding and COIBar–RFLP (Table 1). DNA samples were deposited at the Department of Biological, Geological, and Environmental Science, Section of Animal Biology, in Catania, Italy. A reference COI-barcode library was constructed using COI sequences of the examined species retrieved from GenBank and/or BOLD.

### 2.2. DNA Extraction

Total genomic DNA was extracted and purified from the samples using the DNeasy tissue kit (Qiagen, Hilden, Germany) following the manufacturer’s instruction. The quality of the extracted DNA was analyzed in 0.8% agarose gel and the quantity of the DNA was determined in NanoDrop ND-1000 spectrophotometer (NanoDrop Technologies, Wilmington, DE, USA), by measuring the absorbance at 260 nm. The extracted DNA was stored at −20 °C for further study.

### 2.3. COI Barcode Amplification and Sequencing

COI sequences were obtained using the primer combination of universal primers VF2_t1-50 TGTAAAACGACGGCCAGTCAACCAACCACAAAGACATTGGCAC-30 and FishR2_t1-50 CAGGAAACAGCTATGACACTTCAGGGTGACCGAAGAATCAGAA-30 (described in Ivanova et al. [46] with M13 tails (M13F: TGTAAAACGACGGCCAGT and M13R: CAGGAAACAGCTATGAC) [47] to improve the sequencing quality of the PCR products. PCR amplification was performed in 50 μL total reaction volume. Each reaction contained 0.5 μM of each primer, 0.2 μM dNTPs, 1.5 μM MgCl2, 1X PCR buffer, 1 U of Taq Polymerase (Invitrogen), and 50–100 ng of genomic DNA. The PCR conditions for amplification of were as follows: initial denaturation at 95 °C for 5 min, followed by denaturation at 95 °C (30 s), annealing at 50 °C (45 s) and the extension at 72 °C (60 s) repeated for 30 cycles, and by a final extension at 72 °C for 10 min. Negative controls were included in all PCR runs to ascertain that no cross-contamination occurred. Double stranded products were checked with agarose gel electrophoresis and purified with the QIAquick PCR purification kit (Qiagen, Hilden, Germany). Subsequently, the sequencing of amplified products was performed both for forward and reverse directions using an ABI Prism 3100 automated sequencer (Applied Biosystems, Monza, Italy). Sequences were carefully checked and deposited in GenBank (http://www.ncbi.nlm.nih.gov/genbank/ (accessed on 27 March 2022) under accession numbers reported in Table 1. The chromatograms obtained were assembled and checked by eye. Edited sequences were aligned using the online version of MAFFT v.7 [48] and the alignment was manually revised in BioEdit (http://www.mbio.ncsu.edu/bioedit/bioedit.html (accessed on 27 March 2022). Ambiguous extremities of the sequences were trimmed after alignment. The obtained sequences were carefully checked for the presence of nuclear mitochondrial pseudogenes or NUMTs (nuclear mitochondrial DNA sequences), which could be easily coamplified with orthologous mtDNA sequences [49]. The obtained sequences were searched against GenBank (http://www.ncbi.nlm.nih.gov (accessed on 27 March 2022)) using the nucleotide Basic Local Alignment Search Tool (BLAST), megablast algorithm, and the top species match was recorded. A sequence similarity of at least 98%, with a 100% query coverage, was used to designate potential species identification for the COI gene [29]. An unrooted K2P distance Neighbour-Joining (NJ) tree was generated in MEGA X software [50] to provide a graphical representation of the clustering pattern across different species. The robustness of the tree was evaluated using bootstrap analysis with 1000 iterations [51].

### 2.4. COIBar-RFLP

The “Remap” program (http://emboss.bioinformatics.nl (accessed on 17 March 2022) was used to select suitable restriction enzyme to produce RFLP patterns of investigated species. On the basis of this analysis some restriction enzymes were selected for carrying out the COI-RFLP analysis: *Hind*III, *Alu*I, *Mbo*I, *Hinf*I, *Msp*I (New England Biolabs, Inc., Ipswich, MA, USA). The selected endonucleases were used to digest the COI barcode amplicons of samples obtained from *E. encrasicolus*, *S. pilchardus*, *A. minuta*, *N. tangkahkeii*, *P. fluviatilis*, *L. niloticus* and *Pangasianodon hypophthalmus* (found in one product labeled as *P. fluviatilis*) as reference sample. All the acquired fish products were tested by COIBar-RFLP. The digestion reaction was carried out using a total volume of 12 mL containing 1 mL of enzyme buffer, 1 mL of PCR products and 0.5 mL of each endonuclease (20 U each). The reaction mixture was incubated in a water bath for 1 h at 37 °C. The resulting fragments were separated by electrophoresis on a 3% agarose gel. The sizes of the resulting DNA fragments were estimated by comparison with Trackit 100-bp ladder (Invitrogen, Carlsbad, CA, USA).

## 3. Results

### 3.1. COI Barcode

A fragment of about 651 bp of COI sequences was obtained from 30 tissue samples of seafood products. No insertions, deletions or stop codons were observed in all sequences. The lack of stop codons and the length of the amplified sequences suggest that they are functional mitochondrial COI sequences and that NUMTs (nuclear DNA sequences originating from mitochondrial DNA sequences) were not sequenced (vertebrate NUMTS are generally smaller than 600 bp [49]. The sequences obtained were compared with the sequences retrieved from NCBI GenBank to confirm the species using BLAST option. The percent identity between our COI query sequences and their top-match sequences ranged from 98.67–99.84 with 100% of sequence coverage (Table 1). BLAST search showed 7 cases of misdescription: 3 samples labeled as “bianchetto” (yuveniles of *E. encrasicolus* and *S. pilchardus*) were substituted by *N. tangkahkeii* (2 samples) and *A. minuta* (1 sample); 3 samples labeled as “persico reale” (*P. fluviatilis*) were substituted by *L. niloticus* and 1 sample labeled as “persico reale” was substituted by *P. hypophthalmus*.

**Figure 1:**
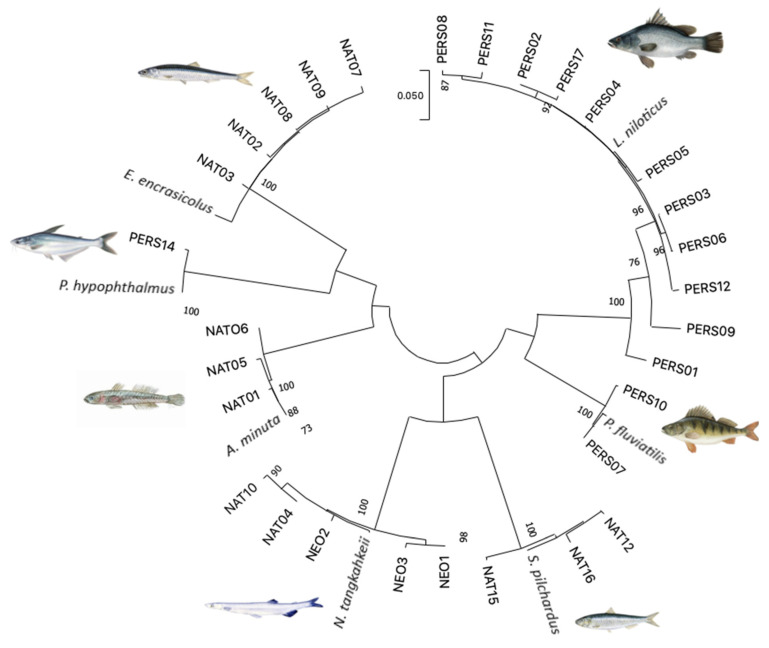
Circular unrooted Neighbour-joining tree (created using MEGA X, [Kumar et al. 2018]) of COI sequences from seafood products examined in this study and reference sequences for each species targeted from GenBank (*A. minuta*, KM077808; *E. encrasicolus*, KU056696; *S. pilchardus*, MG729586; *L. niloticus*, MK216590; *N. tangkahkeii*, OL49421; *P. fluviatilis*, KT716364; *P. hypophthalmus*, MH119967). Scale represents numbers of substitutions per site. Numbers at the nodes show bootstrap values greater than 70%.

The circular NJ tree built using the 7 reference sequences retrived from NCBI GenBank and 30 COI sequences of seafood product samples obtained in the present study (Figure 1), revealed that the sequences of each species clustered together. Each node in the tree was supported by 100% bootstrap value.

### 3.2. COIBar-RFLP Strategy

The endonucleases with the position of restriction sites and the length of expected fragments in each of the tested species are shown in Table 2.

**Figure 2:**
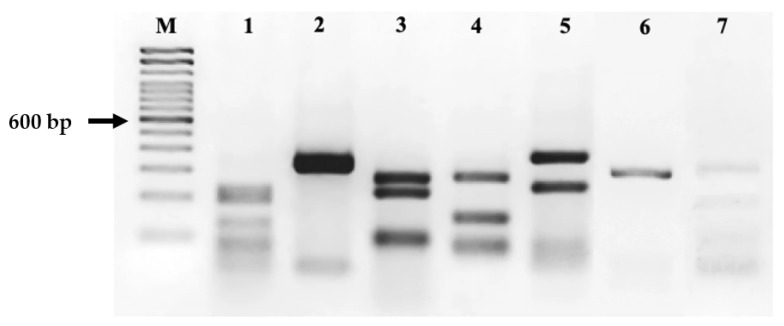
COIBar-RFLP profile of seven seafood species using *Msp*I. Lane M: 100 bp Marker. (**1**) *A. minuta*; (**2**) *E. encrasicolus*; (**3**) *N. tangkahkeii*; (**4**) *L. niloticus*; (**5**) *P. fluviatilis*; (**6**) *S. pilchardus*; (**7**) *P. hypophthalmus*.

The COI sequences of each species were analyzed and the *Msp*I enzyme was selected based on the restriction sites pattern returned by “Remap” program. The restriction digestion performed for the amplified PCR products has produced different RFLP profile for each examined species. The PCR amplification and subsequent *Msp*I digestion shows concordant results and the representative gel for one individual from each species were shown in Figure 2.

As illustrated in Figure 2, the restriction sites yielded three fragments of about 250, 200, and 130 bp in *A. minuta*; two fragments of about 350 and 300 bp in *E. encrasicolus*; three fragments of about 280, 220 and 110 bp in *N. tangkahkeii*. Three fragments of about 290, 150 and 100 bp were detected for *L. niloticus*, and two fragments of about 390 and 250 bp for *P. fluviatilis*. A fragment of about 280–290 bp was obtained for *S. pilchardus* and finally three fragment of 300, 190 and 90 bp were detected for *P. hypophthalmus*. The specific restriction pattern was obtained for each acquired product confirming the sequencing results.

## 4. Discussion

The results obtained by COIBar-RFLP analysis, indicated that the restriction enzyme *Msp*I produced a differential pattern of restriction useful to discriminate all species contained in products sold as “bianchetto”, “rossetto” and European perch fillets. The obtained molecular authentication of species allowed to highlight that in 28% of samples, the species don’t match with the species declared on the label. In particular, the juveniles of *S. pilchardus* and *E. encrasicolus* or “bianchetto” was substituted by *N. tangkahkeii* or icefish and by *A. minuta* or “rossetto” in the 8% and 4% of samples, respectively. The fillets of *P. fluviatilis*, the European perch, were substituted by *L. niloticus,* the Nile perch, and *P. hypophthalmus*, the striped catfish, in 12% and 4% of samples, respectively. These results add new information to those obtained in previous investigation carried out in Northern and Central Italy [52,53,54] and demonstrate that after 10 year the level of mislabeling observed is high.

The late-larval and juvenile stages, up to 4 cm in length, of Clupeid fishes are diaphanous, devoid of pigmentation and for this reason they are referred in Italy as “bianchetto” (whitish); this term collectively describes mainly the European pilchard or sardine (*S. pilchardus*), but also the European anchovy (*E. encrasicolus*) and sprat (*Sprattus sprattus*) [55]. However, the Italian Ministerial Decree (MD) n.19105 22 September 2017 of the Italian Ministry of Agricultural, Food and Forestry Policies, reserves the term “bianchetto” only to the juveniles of *S. pilchardus*; instead, the term “rossetto” is used in Italy to indicate the reddish or whitish juveniles and the adult of the transparent goby, *A. minuta*, a small pelagic species characterized by the persistence in adulthood of several larval features caused by the earlier onset of sexual maturity in the juvenile phase [56]. The European Regulations have included the fishing of juvenile forms, in the “special forms of fishing” of interest for local artisanal fisheries. “bianchetto” and “rossetto” are caught in Italy along the Ligurian, Adriatic and Tyrrhenian coasts [56] and around the coast of Sicily [57] during the winter and, even if sold at high prices (20–40 euros/kilo), they are very valued by consumers. However, since the entry into force of the Council Regulation (EC) No 1967/2006, concerning management measures for the sustainable exploitation of fishery resources in the Mediterranean Sea, the “special forms of fishing” have been banned with few exemption for “rossetto” [58]. These restrictive measures on fishing together with the globalization of the fish trade, have opened the market to the arrival of species from foreing fishery areas, of less commercial value, which filled the void left by the banned species traditionally consumed in the Mediterranean area: this was the case of *Neosalanx* species or icefish, small freshwater fish species, widely imported in Italy from China as frozen product [59,60]. The fraudulent substitution of “bianchetto” by icefish has been already reported in Italy by Armani et al. [53] who used a fragment of the mitochondrial cyt*b* gene as molecular marker and by Guerriero et al. [54] who applied a PCR-RFLP analysis of a partial fragment of the mitochondrial 16S rRNA gene. Two species of *Neosalanx* were detected to be sold as “bianchetto”: *N. tahiuensis* [53] in Northern Italy cities and *N. tangkahkeii* [54] in Central Italy; our results confirm that *N. tangkahkeii* was the species sold as “bianchetto” in Southern Italy. As far as the *Neosalanx* species, it should be noted that the molecular phylogeny of 15 icefish species carried out by Zhang et al. [61] based on complete mtDNA cyt*b* sequences, highlighted that *N. tangkahkeii*, *N. taihuensis*, and *N. pseudotaihuensis* are synonyms and that *N. tangkahkeii* is the valid species first named among the three species. However, they are currently accepted as separated taxon in the FishBase database (www.fishabase.org, version 02/22 (accessed on 19 May 2022). Focusing our attention on the European perch, this freshwater fish species is highly valued in Europe as shown by the high selling price of fillets which are frequently prone to substitution and mislabeling. In Italy, cases of species substitution for fillets of *P. fluviatilis* have been reported in Northern and Central Italy [52] North-Eastern regions [62], Apulia [63,64] and Sardinia [65]. The species more frequently used in place of the European perch are the Nile perch (*L. niloticus*) and the striped catfish (*P. hypophtalmus*); less frequently have been found the giant pangasius (*P. sanitwongsei*); some species belonging to the genus *Paralichthys*, a flounder that has a low market value [62,64], the European pikeperch (*Stizostedion lucioperca*) and the sunshine bass, an hybrid between the striped bass (*Morone chrysops*) and white bass (*M. saxatilis*) [66]. Our results confirm that the fillets of *L. niloticus* and *P. hypophtalmus* are used as surrogate of *P. fluviatilis* in Southern Italy regions.

The damage to consumer deriving from the practice of species substitution of high economic value with species of low value, is frequently only economic. However, implications for human health have also been highlighted. Primarily, the attention has been focused on the missing information about the catch area and the production system of the cryptic species due to intentional mislabeling. However, it is known that both *N. tangkachkeii*, *L. niloticus* and *P. hypophthalmus* come from highly polluted freshwater basins of extra-European countries that pay little attention and no control to the level of pollutant bioaccumulation in edible fish. In this context, classic and emerging contaminants of aquatic environment have been widely detected in fish living in Chinese lakes (Chaohu, Taihu) such as icefish *N. tangkachkeii* and *N. taihuensis* [67,68] and the biomagnification of polyfluoroalkyl substances, mercury, organochlorine pesticides in Nile Perch living in the open waters of African lakes, has been found to pose health risk to the consumers [69,70,71,72]. Furthermore, very recent investigations [73,74] documented high contamination of liver and muscle of *P. hypophthalmus* farmed in Asian Countries highlighting the severe threat for the human health. The abuse of various chemicals and antimicrobial agents represent also a major concern related to the hidden use of Asian aquaculture products such as pangasius fish [75]. In addition to the concerns by pollutant contamination, life-threatening anaphylactic reactions to specific fish allergens are another major issue related to the intentional mislabeling of seafood products. The case of a patient with immediate-type allergy to *L. niloticus* indicating species-specific sensitization towards potential allergens of this species, other than parvalbumin (the most frequent allergen in fish) has been reported [76]. A clinical case of monosensitivity to pangasius was also observed in a patient with oral allergy syndrome [77] and new cat-fish allergens have been identified using a well-characterized cohort of fish-allergic pediatric patients [78].

Based on the above considerations, the authentication of fish species in seafood products is imperative both to prevent threats to human health and to fight food frauds. However, the standardization of the methodology to be used for species identification is also pivotal to face the food safety and quality concerns in a global food market. In this regard, the DNA based technologies have proven to be very effective as food fraud detection tools [13,14] and the methodology of DNA barcoding based on the cytochrome oxidase I sequences is the most used one [12,33,34,35,79]. In the last decade new techniques such as Real Time-PCR, Single Nucleotide Polymorphisms (SNPs), Forensically Informative Nucleotide Sequencing (FINS), Loop-mediated isothermal amplification (LAMP), Droplet Digital PCR (ddPCR), High Resolution Melting Analysis (HRMA), Next Generation Sequencing (NGS) metabarcoding, have been proposed by researchers with the aim of optimizing time, costs and effectiveness of species authentication in multi-species fish products [80,81,82,83]. Although these techniques have been also combined with COI barcoding by providing promising approaches for high throughput species discrimination in processed seafood [84,85,86], traditional mitochondrial DNA-based methods and the PCR-RFLP in particular is still employed due to the advantages offered such as relatively cheapness, lack of technical over-complication, suitability for routine analyses [85]. Despite PCR-RFLP has some disadvantages related to intraspecific variability and the need to select appropriate enzymes it is a well-established and broadly accepted method in seafood authentication. In this context, our results obtained in this and previous studies through COIBar-RFLP analysis, confirm the effectiveness of this molecular approach as demonstrated by the increasing number of successfully discriminated species: so far, a total of 35 fish species has been identified in processed products [11,42,43,44,45]. Interestingly, it should be noted that by coupling COI barcode and RFLP the intraspecific variability in *E. encrasicolus* has been also detected [42].

## 5. Conclusions

In our study the comparison for each sample of the results obtained from the COI sequencing and from the restriction pattern, confirmed the efficacy of COIBar-RFLP for species authentication. Although COI sequences could provide more information for species identification, the COIBar-RFLP takes advantage of the high number of COI sequences available in public database and it is in turn advantagious in the speed of sample processing and cost-effectiveness as it avoids the expense of sequencing. However, building a database of COIBar-RFLP pattern to be used as reference, represents the future per-spective of this methodology which could be a good candidate to become a cost-effective and standardized solution for large-scale screening of seafood species authentication also by operator without specific skills.

## Figures and Tables

**Table 1:** Samples examined in this study.

Sample Code	Declared Species	GenBank	Species Matched by BLAST	Matched GenBank	% Identity
		Accession N°		Accession from BLAST	100% Coverage
NEO01	“icefish” (*Neosalanx tangkahkeii)*	ON242378	*Neosalanx tangkahkeii*	OL494212	99.23
NEO02	“icefish” *(Neosalanx tangkahkeii)*	ON242379	*Neosalanx tangkahkeii*	KP170510	99.20
NEO03	"icefish” (*Neosalanx* spp.)	ON242380	*Neosalanx tangkahkeii*	OL494212	98.77
**NAT01**	**“bianchetto”**	**ON242381**	***Aphia minuta***	**KM077808**	**99.69**
NAT02	“bianchetto”	ON242382	*Engraulis encrasicolus*	MG740790	99.54
NAT03	“bianchetto”	ON242383	*Engraulis encrasicolus*	MG729571	99.54
**NAT04**	**“bianchetto”**	**ON242384**	***Neosalanx tangkahkeii***	**KP170510**	**99.37**
NAT05	“rossetto”	ON242385	*Aphia minuta*	KM077808	99.54
NAT06	“rossetto”	ON242386	*Aphia minuta*	KM077814	99.67
NAT07	“bianchetto”	ON242387	*Engraulis encrasicolus*	KU056679	99.39
NAT08	“bianchetto”	ON242388	*Engraulis encrasicolus*	MG729554	99.84
NAT09	“bianchetto”	ON242389	*Engraulis encrasicolus*	MG729554	99.68
**NAT10**	**“bianchetto”**	**ON242390**	***Neosalanx tangkahkeii***	**KP170510**	**98.89**
NAT12	“bianchetto”	ON242391	*Sardina pilchardus*	MG729586	99.69
NAT15	“bianchetto”	ON242392	*Sardina pilchardus*	MG729588	99.39
NAT16	“bianchetto”	ON242393	*Sardina pilchardus*	EF609451	99.08
**PERS01**	**“persico reale”**	**ON247419**	***Lates niloticus***	**KT193061**	**99.54**
**PERS02**	**“persico reale”**	**ON247420**	***Lates niloticus***	**MN893181**	**99.23**
PERS03	“persico del Nilo”	ON247421	*Lates niloticus*	MN893181	99.54
PERS04	“persico del Nilo”	ON247422	*Lates niloticus*	MK216590	99.68
PERS05	“persico"	ON247423	*Lates niloticus*	MK216590	99.35
PERS06	“persico”	ON247424	*Lates niloticus*	MN893181	99.38
PERS07	“persico reale”	ON247425	*Perca fluviatilis*	AP018422	99.68
PERS08	“persico”	ON247426	*Lates niloticus*	MK216590	99.03
**PERS09**	**“persico reale”**	**ON247427**	***Lates niloticus***	**OL804282**	**99.68**
PERS10	“persico reale”	ON247428	*Perca fluviatilis*	MG969738	99.67
PERS11	“persico del Nilo”	ON247429	*Lates niloticus*	MK216590	98.87
PERS12	“persico”	ON247430	*Lates niloticus*	MK216590	99.03
**PERS14**	**“persico reale”**	**ON247431**	***Pangasianodon hypophthalmus***	**MH119967**	**99.68**
PERS17	“persico”	ON247432	*Lates niloticus*	MN893181	98.62

In bold mislabeling.

**Table 2:** COIBar-RFLP expected profiles (in base pair of DNA fragments) of the investigated species in this study. ND, not digested.

	*Aphia minuta*	*Engraulis encrasicolus*	*Sardina pilchardus*	*Neosalanx tangkahkeii*	*Lates niloticus*	*Perca fluviatilis*	*Pangasius hypophthalmus*
*Hind*III	200/400	ND	ND	ND	ND	ND	ND
*Alu*I	180/100/200	205/270/100	220/270/150	250/250/100	220/200/100	200/210/140	100/450/150
*Mbo*I	200/450	100/320/330	130/180	100/430	100/480	150/450	ND
*Hinf*I	260/150/250	260/390	ND	190/200/230	260/390	240/350	ND
*Msp*I	320/290/100	300/280	280/100	180/100/210	300/170/150	240/350	100/300/200/100

## Data Availability

The data presented in this study are available on request from the corresponding author.
